# Multi-Frequency Signal Detection Based on Frequency Exchange and Re-Scaling Stochastic Resonance and Its Application to Weak Fault Diagnosis

**DOI:** 10.3390/s18051325

**Published:** 2018-04-25

**Authors:** Jinjun Liu, Yonggang Leng, Zhihui Lai, Shengbo Fan

**Affiliations:** 1 School of Mechanical Engineering, Tianjin University, Tianjin 300350, China; liujj@tju.edu.cn (J.L.); fanshb@tju.edu.cn (S.F.); 2 School of Mechatronics Engineering, Nanchang University, Nanchang 330031, China; laizh@ncu.edu.cn

**Keywords:** signal processing, stochastic resonance, frequency re-scaling, frequency exchange, multi-frequency signal detection

## Abstract

Mechanical fault diagnosis usually requires not only identification of the fault characteristic frequency, but also detection of its second and/or higher harmonics. However, it is difficult to detect a multi-frequency fault signal through the existing Stochastic Resonance (SR) methods, because the characteristic frequency of the fault signal as well as its second and higher harmonics frequencies tend to be large parameters. To solve the problem, this paper proposes a multi-frequency signal detection method based on Frequency Exchange and Re-scaling Stochastic Resonance (FERSR). In the method, frequency exchange is implemented using filtering technique and Single SideBand (SSB) modulation. This new method can overcome the limitation of "sampling ratio" which is the ratio of the sampling frequency to the frequency of target signal. It also ensures that the multi-frequency target signals can be processed to meet the small-parameter conditions. Simulation results demonstrate that the method shows good performance for detecting a multi-frequency signal with low sampling ratio. Two practical cases are employed to further validate the effectiveness and applicability of this method.

## 1. Introduction

Various types of rotating machinery, such as electric generators, water turbines, centrifugal compressors, pumps, etc., have played an important role in industry. During the design, installation, application, and other processes, some potential risks of fault may exist in the rotating machinery system, which can result in a serious threat to the safe operation of equipment. Therefore, researchers have produced a mountain of research on the health monitoring and fault diagnosis of rotating machines [1,2,3]. In particular, the detection of incipient faults has become the focus of a number of studies of fault diagnosis. Some methods, such as wavelet transform [4,5], empirical mode decomposition [6], and nonlinear dynamic theory [7,8], have been studied in depth and widely for weak fault feature extraction in many practical situations. The weak signal can be simply amplified without distortion by a linear amplifier. However, while the weak fault signal submerged in heavy background noise is amplified by a linear amplifier, the background noise is also amplified in the same proportion. The Signal-to-Noise Ratio (SNR) of the amplified signal is not improved. Therefore, the weak fault characteristic signal is still submerged in the background noise and cannot be identified.

Stochastic Resonance (SR) is an unusual nonlinear phenomenon which can be used to enhance and detect weak signals embedded in heavy background noise. SR theory has been of wide concern since its discovery by Italian physicists Benzi, Sutera and Vulpiani [9] in the 1980s and it has been verified experimentally in the Schmitt trigger circuit [10] and the bidirectional ring laser [11]. Soon after, the theory of SR in the adiabatic limit was proposed [12,13,14]. To date, SR theory has been studied and applied in many fields. Collins [15] discussed the subthreshold SR phenomenon in the FitzHugh-Nagumo (FHN) neuronal model, and Stocks [16] studied the suprathreshold SR phenomenon in multilevel threshold systems. The two SR phenomena were realized by the conventional method of introducing noise into the threshold systems. Another way to realize SR phenomenon is the method of tuning nonlinear system parameters [17], which is more practical than the method of adding noise to the nonlinear systems for signal processing [18]. Bates et al. [19] found an SR phenomenon in an intracellular associative genetic perceptron, which manifests itself by the improvement of response in efficiency after the learning event under the optimal level of noise. The SR techniques have also been applied to image de-noising and enhancement [20,21]. In the field of signal transmission, Duan et al. [22] designed a bi-stable dynamic receiver to detect binary modulated signals and observed non-conventional SR phenomena, such as residual ASR and short-time SR effects. Qi et al. [23] studied a modified adaptive stochastic resonance to detect fault information from the power supply. While in the field of mechanical fault diagnosis, some weak fault signal detection methods based on SR have been investigated to extract the incipient fault features from mechanical vibration signals [24,25,26].

Due to the limitation of strict small-parameter conditions (the amplitude and frequency of periodic signal and noise intensity are far less than 1), via SR theory, it is difficult to detect the mechanical fault signals which usually have a large-parameter frequency (greater than 1 Hz). In order to deal with the large-parameter problem in practical engineering applications, a variety of solutions have been proposed, such as Frequency Re-scaling Stochastic Resonance (FRSR) [25,27], Parameters Normalized Stochastic Resonance (PNSR) [28], Modulated Stochastic Resonance (MSR) [29], Frequency Shifted and Re-scaling Stochastic Resonance (FSRSR) [30]. Actually, the main aim of these methods is to transform the large-parameter signal into a small-parameter signal which satisfies the small-parameter conditions. Then it can achieve detection of the large-parameter signal by SR theory. Although these methods have been applied to engineering applications successfully, they also have their own limitations. For instance, FRSR and PNSR require a higher sampling ratio (greater than 50) which is the ratio of the sampling frequency to the frequency of the target signal. The theory of Double SideBand (DSB) modulation used in MSR, may result in a signal superposition problem which will affect the results of detection.

In addition, it requires not only detection of the fault characteristic frequency, but also the identification of its second and higher harmonics in fault diagnosis and condition monitoring systems. To date, researchers have studied the multi-frequency fault signal detection based on SR. In the presence of colored noise, Xu et al. [31] studied multi-frequency signal processing and recovery via SR with tuning system parameters. Jiao et al. [32] studied the detection of the multi-frequency signal embedded in *α* stable noise by means of parameter-induced SR and parameter compensation. Shi et al. [33] proposed a method for multi-frequency signal detection based on orthogonal wavelet transform and SR. In the paper, the original signal is decomposed into different scales by orthogonal wavelet transform, and then some decomposed components containing the target signals are respectively detected by FRSR. A multi-frequency weak signal detection method based on discrete wavelet transform and parameter compensation band-pass multi-stable SR is proposed in the paper [34] by Han. To detect the multi-frequency weak fault signal of rolling bearings, Guo et al. [35] studied a method based on the multi-segmentation of data and cascaded SR. These methods for detecting multi-frequency fault signal, however, still have such problems as relatively high sampling frequency and a large amount of sampling points. Therefore, they are still not convenient for practical application. It is necessary to solve the problems further to make SR more suitable for multi-frequency signal detection.

In this paper, a multi-frequency signal detection method based on Frequency Exchange and Rescaling Stochastic Resonance (FERSR) is proposed. In the method, frequency exchange is first carried out based on filtering technique and SSB modulation. For a multi-frequency signal, it is necessary to design a multi-band filter bank for extracting the signal at a different characteristic frequency. After that, the frequencies of the processed signals are rescaled. Through classical stochastic resonance and frequency recovery, the characteristic frequency information of the original signal can finally be read from a three-dimensional frequency spectrogram.

The paper is organized as follows. In Section 2, the classical stochastic resonance theory, frequency rescaling stochastic resonance theory and their existing problems are introduced. In Section 3, this paper elaborates the frequency exchange method and proposes a signal detection method based on frequency exchange and rescaling stochastic resonance. In Section 4, a filter bank is designed to extract first the multi-frequency signal. Additionally, frequency exchange is introduced by Single SideBand (SSB) modulation theory. Finally, the multi-frequency signal detection method based on FERSR is proposed. The simulation analysis, experimental verification, and discussion on this method are given in Section 5. Conclusions are provided in the last section.

## 2. Basic Theory

### 2.1. Classical Stochastic Resonance (CSR)

The mechanism of CSR is simple to explain. Assuming that an overdamped Brownian particle moving in a symmetric double-well potential is driven by fluctuation forces. For a convenient description, in the presence of noise and periodic forcing, the overdamped motion of a Brownian particle in a bi-stable potential can be described by the Langevin equation,

(1) $$\frac{dx}{dt}=ax-bx^{3}+s\left(t\right)+n\left(t\right)$$

where *a* and *b* are barrier parameters with positive real values. s(t) is a periodic signal with amplitude *A* and frequency $f_{0}$, $s\left(t\right)=A\cos\left(2\pi f_{0}t\right)$; n(t) denotes a zero-mean, Gaussian white noise with noise intensity *D* and autocorrelation function 〈n(t)n(t−τ)〉=2Dδ(τ).

For small amplitudes, the response of the system to the periodic input signal can be written as [13]

(2) $$\langle x\left(t\right)\rangle =\bar{x}\cos\left(2\pi f_{0}t-\bar{\phi }\right)$$

where $\bar{x}$ and $\bar{\phi }$ are the amplitude and a phase lag respectively. The approximate expressions of the amplitude $\bar{x}$ is

(3) $$\bar{x}=\frac{A{\langle x^{2}\rangle }_{0}}{D}\frac{r_{k}}{\sqrt{r_{k}^{2}+\pi^{2}f_{0}}}$$

where ${\langle x^{2}\rangle }_{0}$ is the *D*-dependent variance of the stationary unperturbed system (*A* = 0). $r_{k}$ is Kramers rate and can be written as

(4) $$r_{k}=\frac{a}{\sqrt{2}\pi }\exp\left(-\frac{a^{2}}{4bD}\right)$$

After rewriting Equation (3), the approximate amplitude amplification value *K* of the response of the SR system is given by

(5) $$K=\bar{x}/A=\frac{{\langle x^{2}\rangle }_{0}}{D}\frac{r_{k}}{\sqrt{r_{k}^{2}+\pi^{2}f_{0}}}=\frac{a}{bD\sqrt{1+\frac{2\pi^{4}f_{0}^{2}}{a^{2}}\exp\left(\frac{a^{2}}{2bD}\right)}}$$

**Figure 1:**
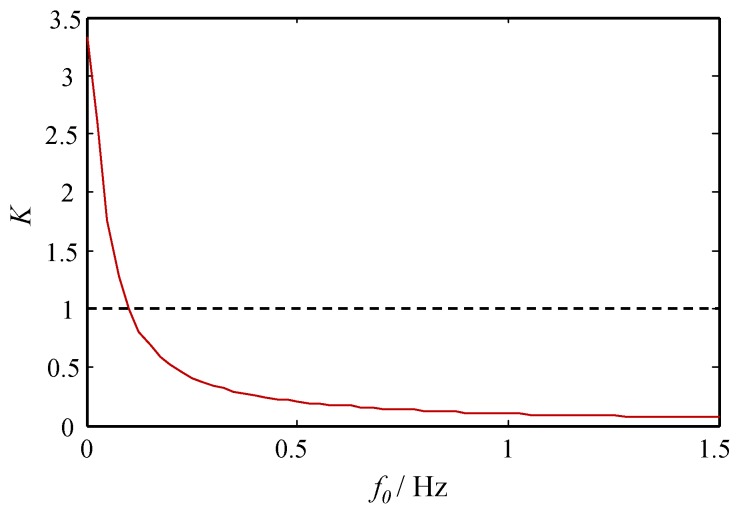
The response amplification value *K* of the classical stochastic resonance (CSR) system versus the frequency $f_{0}$.

As demonstrated in Figure 1, the amplitude amplification value *K* decreases monotonically with the frequency of the input periodic signal. When the frequency is a large parameter, the output signal is not enhanced but weakened, which is caused by the small-parameter conditions of CSR theory.

### 2.2. Frequency Re-Scaling Stochastic Resonance

In order to detect large-parameter frequency signals, Leng et al. [25,27] proposed the Frequency Re-scaling Stochastic Resonance (FRSR). In the FRSR method, the sampling frequency $f_{s}$ and the large-parameter characteristic frequency $f_{0}$ are rescaled in proportion by introducing the scale factor *R* (>1). Then $f_{0}$ is transformed into a small-parameter frequency which satisfies the small-parameter conditions in order to realize the detection of the large-parameter fault signals based on CSR. The rescaled sampling frequency is

(6) $$f_{sr}=f_{s}/R$$

The rescaled characteristic frequency is

(7) $${f^{\prime }}_{0}=f_{0}/R$$

Correspondingly, the time is rescaled as

(8) $$t^{\prime }=Rt$$

For the scale-transformed signal, Equation (1) can be rewritten as

(9) $$\frac{dx}{dt^{\prime }}=ax-bx^{3}+A\sin\left(2\pi {f^{\prime }}_{0}t^{\prime }\right)+n\left(t^{\prime }\right)$$

When the frequency ${f^{\prime }}_{0}$ satisfies the small-parameter conditions, the characteristic signal can usually be detected by Equation (9). However, it is only when the sampling ratio λ is relatively high that the FRSR method has a good detection performance. The sampling ratio λ is the ratio of sampling frequency $f_{s}$ to the frequency $f_{0}$, i.e., $\lambda =f_{s}/f_{0}$. Paper [27] demonstrates that the sampling ratio λ should be greater than 50. For a low sampling ratio, it is usually impossible to find the appropriate *R* to ensure both requirements that the rescaled frequency ${f^{\prime }}_{0}$ meets the small-parameter conditions and the solution of Equation (9) is convergent. It should be noted that the Runge-Kutta method is used to solve Equation (9). The step length h of the Runge-Kutta method is also directly related to the convergence of the solution. And the step length is given by $h=1/f_{sr}=R/f_{s}$, hence, the coefficient *R* affects the convergence of the solution directly.

## 3. Frequency Exchange and Re-Scaling Stochastic Resonance (FERSR)

In order to overcome the constraint of sampling ratio λ of FRSR and detect higher-frequency signals, Frequency-Information-Exchange Stochastic Resonance (FIESR) was studied in [36]. In that paper, the frequency information of a higher-frequency signal is exchanged with that at low frequency in the frequency spectrum obtained by Fast Fourier Transformation (FFT). Then the signal detection can be achieved by FRSR. However, FIESR still has some difficulty in practical application, because the accuracy of frequency information extraction is affected by the leakage in the frequency spectrum. The spectrum leakage is caused by incoherent sampling and a finite number of samples. In view of this situation, this section presents a new FERSR method based on filtering technique and SSB modulation in time domain in which, it can facilitate the application of the SR method.

### 3.1. The Principle of Frequency Exchange Based on SSB Modulation

**Figure 2:**
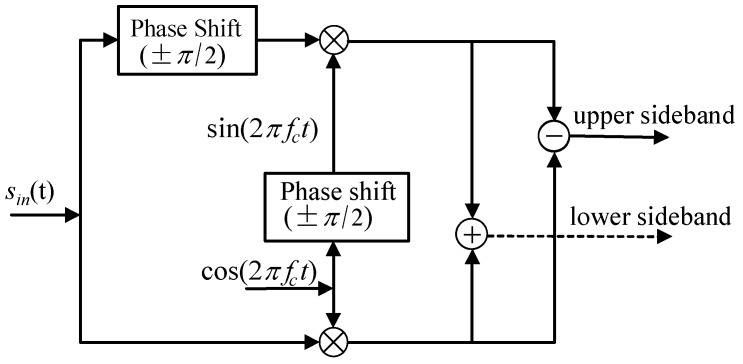
Schematic diagram of single-sideband modulation.

The principle of frequency exchange based on SSB modulation is depicted in Figure 2. Firstly, an input signal $s_{in}\left(t\right)$ and a carrier signal $\cos\left(2\pi f_{c}t\right)$ are shifted in phase by π/2 through Hilbert transform. After that, the upper sideband of the carrier signal and lower sideband of the input signal can be achieved by SSB modulation. The Hilbert transform of a continuous signal s(t) is defined as

(10) $$\overset{\wedge }{s}\left(t\right)=H\left[s\left(t\right)\right]=h\left(t\right)*s\left(t\right)=\int_{-\infty }^{\infty }s\left(\tau \right)\times h\left(t-\tau \right)d\tau =\frac{1}{\pi }\int_{-\infty }^{\infty }\frac{s\left(\tau \right)}{t-\tau }d\tau$$

where H[•] denotes the Hilbert transform operator. The time impulse response of the Hilbert transformer is

(11) $$h\left(t\right)=\frac{1}{\pi t}$$

As shown in Figure 2, the upper-sideband modulation of the input signal $s_{in}\left(t\right)$ is

(12) $$s_{USB}\left(t\right)=s_{in}\left(t\right)\times \cos\left(2\pi f_{c}t\right)-H\left[s_{in}\left(t\right)\right]\times H\left[\cos\left(2\pi f_{c}t\right)\right]$$

and the lower-sideband modulation of $s_{in}\left(t\right)$ is

(13) $$s_{LSB}\left(t\right)=s_{in}\left(t\right)\times \cos\left(2\pi f_{c}t\right)+H\left[s_{in}\left(t\right)\right]\times H\left[\cos\left(2\pi f_{c}t\right)\right]$$

Assuming that the characteristic signal is $m_{t}\left(t\right)=A_{t}\cos\left(2\pi f_{t}t+\varphi_{t}\right)$, the reference low-frequency signal is $m_{l}\left(t\right)=A_{l}\cos\left(2\pi f_{l}t+\varphi_{l}\right)$ and the carrier signal is $\cos\left(2\pi f_{c}t\right)$.

Then the upper sideband modulation of the signal $m_{l}\left(t\right)$ is

(14) $$\begin{array}{cc} m_{l}^{\prime }\left(t\right) & =m_{l}\left(t\right)\times \cos\left(2\pi f_{c}t\right)-H\left[m_{l}\left(t\right)\right]\times H\left[\cos\left(2\pi f_{c}t\right)\right]\\ & =A_{l}\cos\left(2\pi f_{l}t+\varphi_{l}\right)\times \cos\left(2\pi f_{c}t\right)-A_{l}\sin\left(2\pi f_{l}t+\varphi_{l}\right)\times \sin\left(2\pi f_{c}t\right)\\ & =A_{l}\cos\left[2\pi \left(f_{l}+f_{c}\right)t+\varphi_{l}\right] \end{array}$$

and the lower sideband modulation of the signal $m_{t}\left(t\right)$ is

(15) $$\begin{array}{cc} m_{t}^{\prime }\left(t\right) & =m_{t}\left(t\right)\times \cos\left(2\pi f_{c}t\right)+H\left[m_{t}\left(t\right)\right]\times H\left[\cos\left(2\pi f_{c}t\right)\right]\\ & =A_{t}\cos\left(2\pi f_{t}t+\varphi_{t}\right)\times \cos\left(2\pi f_{c}t\right)+A_{t}\sin\left(2\pi f_{t}t+\varphi_{t}\right)\times \sin\left(2\pi f_{c}t\right)\\ & =A_{t}\cos\left[2\pi \left(f_{t}-f_{c}\right)t+\varphi_{t}\right] \end{array}$$

If the carrier frequency is given as $f_{c}=f_{t}-f_{l}$, then Equations (14) and (15) can be expressed as, $m_{l}^{\prime }\left(t\right)=A_{l}\cos\left(2\pi f_{t}t+\varphi_{l}\right)$, $m_{t}^{\prime }\left(t\right)=A_{t}\cos\left(2\pi f_{l}t+\varphi_{t}\right)$. Therefore, the frequencies of $m_{t}\left(t\right)$ and $m_{l}\left(t\right)$ are exchanged. This is the principle of frequency exchange. For a given sampling frequency $f_{s}$, if the sampling ratio of the target signal $m_{t}\left(t\right)$ is less than 50, i.e., $f_{s}/f_{t}<50$, $m_{t}\left(t\right)$ is called the low sampling ratio signal. Through frequency exchange, the amplitude and phase of $m_{t}\left(t\right)$ is exchanged to the reference low frequency $f_{l}$, and the exchanged target signal $m_{t}^{\prime }\left(t\right)$ is obtained. For the sampling frequency $f_{s}$, if $f_{s}/f_{t}>50$, the signal $m_{t}^{\prime }\left(t\right)$ is called the high sampling ratio signal. That is to say, the sampling ratio of the target signal $m_{t}\left(t\right)$ can be increased by frequency exchange. Then, if the reference low frequency $f_{l}$ is compressed by *R*-scale into $f_{l}/R$ which is a small parameter, then $m_{t}^{\prime }\left(t\right)$ will be a small-parameter signal which can be detected by CSR. In this paper, the frequency range (0 Hz, 0.1 Hz) is selected as reference low frequency band for the sampling frequency 5 Hz.

### 3.2. Signal Processing Model Based on FERSR

Usually, there are some errors between the theoretical value and the actual value of the characteristic frequency (e.g., the fault frequency of rolling bearings), which are caused by fabrication errors, disturbance in the process of data acquisition, and other influencing factors. Because of this, the local information around the characteristic frequency has to be extracted rather than the single specific signal at that frequency. Therefore, in order to ensure that the characteristic frequency is exchanged without being missed, some filters with certain bandwidth are used to extract the target signals. In addition, in order to avoid signal superposition during signal processing, it is required that the passband widths of low-pass filter and band-pass filter are the same as the stopband widths of the high-pass filter and the band-stop filter.

**Figure 3:**
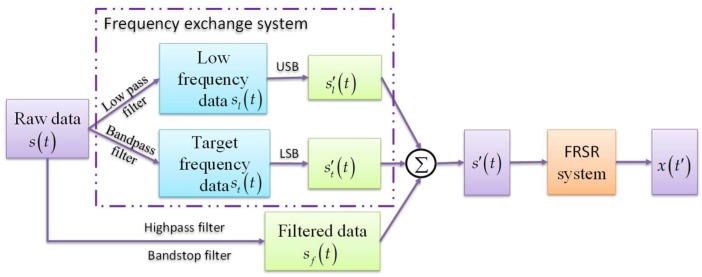
The flow diagram of Frequency Exchange and Re-scaling Stochastic Resonance (FERSR).

Figure 3 shows the signal processing model based on FERSR which includes the following steps: First, for an original data s(t), the local information $s_{l}\left(t\right)$ around the reference low frequency and the local information $s_{t}\left(t\right)$ around the characteristic frequency are extracted respectively through a low-pass filter and a band-pass filter. Meanwhile, the residual information $s_{f}\left(t\right)$ is obtained by filtering out $s_{l}\left(t\right)$ and $s_{t}\left(t\right)$ through a high-pass filter and a band-stop filter correspondingly. Secondly, the frequencies of $s_{l}\left(t\right)$ and $s_{t}\left(t\right)$ are exchanged by the frequency exchange method, then the processed local signals $s_{l}^{\prime }\left(t\right)$ and $s_{t}^{\prime }\left(t\right)$ are obtained. After that, the residual signal $s_{f}\left(t\right)$ is added to the signals $s_{l}^{\prime }\left(t\right)$ and $s_{t}^{\prime }\left(t\right)$, so as to reconstruct the frequency-exchanged signal $s^{\prime }\left(t\right)$ which has the same data length as the original signal s(t). Finally, the response $x\left(t^{\prime }\right)$ of the FRSR system to the input signal $s^{\prime }\left(t\right)$ can be simply obtained.

According to Equation (9), the equation of FERSR can be expressed as

(16) $$\frac{dx}{dt^{\prime }}=ax-bx^{3}+s^{\prime }\left(t^{\prime }\right)$$

where $s^{\prime }\left(t^{\prime }\right)$ denotes the rescaled signal of the frequency-exchanged signal $s^{\prime }\left(t\right)$.

To achieve the goal of detecting the target signal, it is necessary to recover the frequency of the system output $x\left(t^{\prime }\right)$. The frequency recovery involves three steps. The frequency scale of $x\left(t^{\prime }\right)$ is recovered first, which is called inverse frequency re-scaling. Then, a low-pass filter is used to wipe off the interference of the high-frequency components and extract information in the reference low-frequency band which contains the target signal. Finally, the information is moved to its original frequency band though upper sideband modulation.

**Figure 4:**
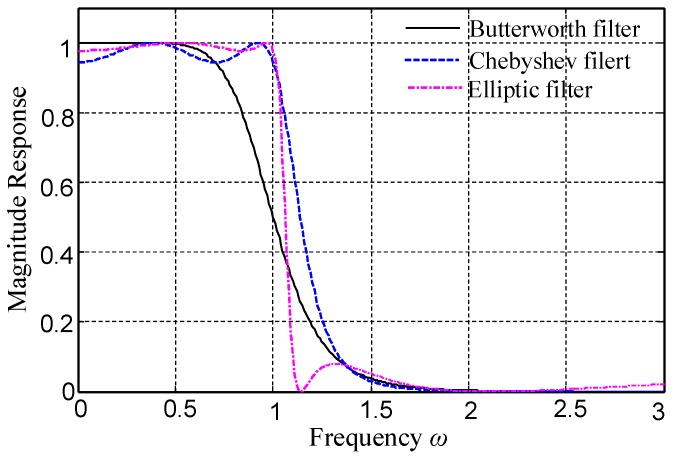
Frequency response characteristics of the Elliptic filter, Chebyshev filter, and Butterworth filter.

It should be noted that, in order to reduce the influence of the transition band of filter, an Elliptic filter is chosen in this paper. As shown in Figure 4, the transition band of the Elliptic filter is narrower than that of the Chebyshev filter and Butterworth filter.

## 4. Multi-Frequency Signal Detection Based on FERSR

In rotating machinery, the fault signals are usually in the form of harmonics. In order to determine the form of fault accurately, it is necessary to detect and analyze the features of the harmonics of the faults. The higher harmonics of some mechanical faults (e.g., those of rolling bearings) are often in the high frequency range. Hence, it is difficult to obtain satisfactory results if a multi-frequency fault signal analysis is performed with a relatively low sampling frequency. Based on FERSR, this section explores the method for extracting multi-frequency signal feature, so as to realize the detection of the first several harmonics of faults with a low sampling ratio.

Assuming that an original signal s(t) has *n* characteristic frequencies and the *i*-th characteristic frequency is $f_{i}$. Two sets of filter banks are designed for the *n* target signals on the basis of their characteristic frequencies. One group is the Band-Pass Filter Bank (*BPFB*) composed of *n* Band-Pass Filters (*BPF*) with passband width *B*, i.e., $BPFB={\left[\begin{array}{ccccc} BPF_{1} & \cdots & BPF_{i} & \cdots & BPF_{n} \end{array}\right]}^{T}$. Similarly, the other is the Band-Stop Filter Bank (*BSFB*) composed of *n* Band-Stop Filters (*BSF*) with stopband width *B*, i.e., $BSFB={\left[\begin{array}{ccccc} BSF_{1} & \cdots & BSF_{i} & \cdots & BSF_{n} \end{array}\right]}^{T}$. The pass band of the *i*-th band-pass filter is the same as the stop band of the *i*-th band-stop filter used in the filter banks, which is $(f_{i}-f_{l},f_{i}-f_{l}+B)$. Here, by *R*-scale re-scaling, the reference frequency $f_{l}$ is compressed into $f_{l}/R$ which is a small parameter. According to the analysis in Section 3.2, only one Low-Pass Filter (*LPF*) and one High-Pass Filter (*HPF*) are needed here to acquire and filter out the information of the original signal around the reference frequency respectively. The pass band of *LPF* and stop band of *HPF* are still (0, *B*).

The local information around each characteristic frequency can be extracted from the original signal s(t) by *BPFB*, i.e.,

(17) $$s_{t}\left(t\right)=s\left(t\right)\;*\;BPFB=s\left(t\right)\;*\;\left[\begin{array}{c} BPF_{1}\\ \vdots \\ BPF_{i}\\ \vdots \\ BPF_{n} \end{array}\right]\;=\;\left[\begin{array}{c} s_{t1}\left(t\right)\\ \vdots \\ s_{ti}\left(t\right)\\ \vdots \\ s_{tn}\left(t\right) \end{array}\right]$$

and by *LPF*, the local information around the reference frequency can be obtained, i.e.,

(18) $$s_{l}\left(t\right)=s\left(t\right)\;*\;LPF$$

After filtering out the local information around each characteristic frequency and the reference frequency by *BSFB* and *HPF*, the residual signal can be obtained, i.e.,

(19) $$s_{f}\left(t\right)=s\left(t\right)\;*HPF*\;BSFB=s\left(t\right)\;*HPF*\;\left[\begin{array}{c} BSF_{1}\\ \vdots \\ BSF_{i}\\ \vdots \\ BSF_{n} \end{array}\right]\;=\;\left[\begin{array}{c} s_{f1}\left(t\right)\\ \vdots \\ s_{fi}\left(t\right)\\ \vdots \\ s_{fn}\left(t\right) \end{array}\right]$$

The obtained local characteristic information is shifted from each characteristic frequency to the reference frequency respectively by lower sideband modulation, that is

(20) $$\left\{ \begin{array}{l} s_{t1}^{\prime }\left(t\right)=s_{t1}\left(t\right)\times \cos\left(2\pi f_{c1}t\right)+H\left[s_{t1}\left(t\right)\right]\times H\left[\cos\left(2\pi f_{c1}t\right)\right]\\ \vdots \\ s_{ti}^{\prime }\left(t\right)=s_{ti}\left(t\right)\times \cos\left(2\pi f_{ci}t\right)+H\left[s_{ti}\left(t\right)\right]\times H\left[\cos\left(2\pi f_{ci}t\right)\right]\\ \vdots \\ s_{tn}^{\prime }\left(t\right)=s_{tn}\left(t\right)\times \cos\left(2\pi f_{cn}t\right)+H\left[s_{tn}\left(t\right)\right]\times H\left[\cos\left(2\pi f_{cn}t\right)\right] \end{array}\right.$$

The information around the reference frequency is shifted to each characteristic frequency respectively by upper sideband modulation, that is

(21) $$\left\{ \begin{array}{l} s_{l1}^{\prime }\left(t\right)=s_{l}\left(t\right)\times \cos\left(2\pi f_{c1}t\right)-H\left[s_{l}\left(t\right)\right]\times H\left[\cos\left(2\pi f_{c1}t\right)\right]\\ \vdots \\ s_{li}^{\prime }\left(t\right)=s_{l}\left(t\right)\times \cos\left(2\pi f_{ci}t\right)-H\left[s_{l}\left(t\right)\right]\times H\left[\cos\left(2\pi f_{ci}t\right)\right]\\ \vdots \\ s_{ln}^{\prime }\left(t\right)=s_{l}\left(t\right)\times \cos\left(2\pi f_{cn}t\right)-H\left[s_{l}\left(t\right)\right]\times H\left[\cos\left(2\pi f_{cn}t\right)\right] \end{array}\right.$$

where the *i*-th carrier frequency is $f_{ci}=f_{i}-f_{l}$. Therefore, *n* sets of frequency-exchanged signal are given by

(22) $$\left[\begin{array}{c} s_{1}^{\prime }\left(t\right)\\ \vdots \\ s_{i}^{\prime }\left(t\right)\\ \vdots \\ s_{n}^{\prime }\left(t\right) \end{array}\right]=\left[\begin{array}{c} s_{t1}^{\prime }\left(t\right)\\ \vdots \\ s_{ti}^{\prime }\left(t\right)\\ \vdots \\ s_{tn}^{\prime }\left(t\right) \end{array}\right]+\left[\begin{array}{c} s_{l1}^{\prime }\left(t\right)\\ \vdots \\ s_{li}^{\prime }\left(t\right)\\ \vdots \\ s_{ln}^{\prime }\left(t\right) \end{array}\right]+\left[\begin{array}{c} s_{f1}\left(t\right)\\ \vdots \\ s_{fi}\left(t\right)\\ \vdots \\ s_{fn}\left(t\right) \end{array}\right]$$

Then, these *n* sets of signals are rescaled and input into the CSR system, the equations of FERSR can be obtained as

(23) $$\left[\begin{array}{c} dx_{1}\left(t^{\prime }\right)/dt^{\prime }\\ \vdots \\ dx_{i}\left(t^{\prime }\right)/dt^{\prime }\\ \vdots \\ dx_{n}\left(t^{\prime }\right)/dt^{\prime } \end{array}\right]=\left[\begin{array}{ccccc} a_{1} & & & & \\ & \ddots & & & \\ & & a_{i} & & \\ & & & \ddots & \\ & & & & a_{n} \end{array}\right]\left[\begin{array}{c} x_{1}\\ \vdots \\ x_{i}\\ \vdots \\ x_{n} \end{array}\right]-\left[\begin{array}{ccccc} b_{1} & & & & \\ & \ddots & & & \\ & & b_{i} & & \\ & & & \ddots & \\ & & & & b_{n} \end{array}\right]\left[\begin{array}{c} x_{1}^{3}\\ \vdots \\ x_{i}^{3}\\ \vdots \\ x_{n}^{3} \end{array}\right]+\left[\begin{array}{c} s_{1}^{\prime }\left(t^{\prime }\right)\\ \vdots \\ s_{i}^{\prime }\left(t^{\prime }\right)\\ \vdots \\ s_{n}^{\prime }\left(t^{\prime }\right) \end{array}\right]$$

where $s_{i}^{\prime }\left(t^{\prime }\right)$ denotes the rescaled signal of the frequency-exchanged signal $s_{i}^{\prime }\left(t\right)$, the rescaled time is $t^{\prime }=tR$, and the system parameters of the *i*-th target signal are $a_{i}$ and $b_{i}$ which can be obtained by parameter optimization algorithms [37].

Using the fourth-order Runge-Kutta method to solve the Equation (23), the results of the target signals processed by FRSR can be expressed as ${\left[\begin{array}{ccccc} x_{1}\left(t^{\prime }\right) & \cdots & x_{i}\left(t^{\prime }\right) & \cdots & x_{n}\left(t^{\prime }\right) \end{array}\right]}^{T}$. After frequency recovery, the recovered signals ${\left[\begin{array}{ccccc} x_{1}\left(t\right) & \cdots & x_{i}\left(t\right) & \cdots & x_{n}\left(t\right) \end{array}\right]}^{T}$ can be obtained. And then the target signals are observed clearly in the three-dimensional frequency spectrogram of the recovered signals ${\left[\begin{array}{ccccc} X_{1}\left(j2\pi f\right) & \cdots & X_{i}\left(j2\pi f\right) & \cdots & X_{n}\left(j2\pi f\right) \end{array}\right]}^{T}$.

## 5. Numerical Simulation and Experimental Verification

### 5.1. Numerical Simulation

In a practical engineering system, the frequencies of target signals are usually arbitrary, or there is a certain mathematical relationship between them. In order to illustrate the effectiveness of the method of multi-frequency signal detection based on FERSR, this section analyzes the simulated multi-frequency signal with several arbitrary characteristic frequencies.

**Figure 5:**
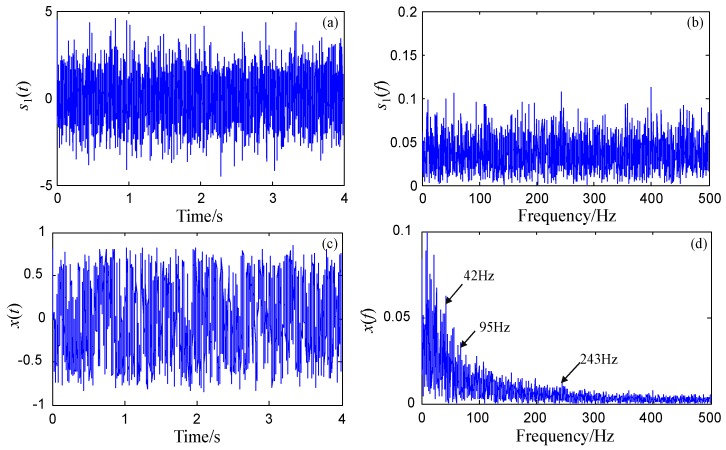
Signal processing based on Frequency Re-scaling Stochastic Resonance (FRSR). (**a**,**b**) are the time-domain waveform and frequency spectrum of the original signal; (**c**,**d**) are time-domain waveform and frequency spectrum of the response of the FRSR system with scale factor *R* = 200.

The original multi-frequency signal $s_{1}\left(t\right)$, composed of three sinusoidal signals and Gaussian white noise, is defined as

(24) $$s_{1}\left(t\right)=A_{1}\times \sin\left(2\pi f_{1}t\right)+A_{2}\times \sin\left(2\pi f_{2}t\right)+A_{3}\times \sin\left(2\pi f_{3}t\right)+n\left(t\right)$$

where n(t) denotes the Gaussian white noise with noise intensity 0.8, and three sinusoidal signals have the same amplitude 0.1 and different frequencies, e.g., $f_{1}=42\;\mathrm{Hz}$, $f_{2}=95\;\mathrm{Hz}$, and $f_{3}=243\;\mathrm{Hz}$. The sampling frequency $f_{s}=1000\;\mathrm{Hz}$ and the number of sampling points N=4000. The time-domain waveform and frequency spectrum of the original signal are shown in Figure 5a,b. It is clear that it is difficult to identify the three signals from the spectrum due to the presence of noise. If the original signal is processed directly by FRSR, as shown in Figure 5d, it is also difficult to find out these signals from the output of the FRSR system, with the parameters *a* = 0.5, *b* = 4, and the scale factor *R* = 200. It should be noted that Figure 5c,d shows the recovered time-domain waveform and frequency spectrum of the FRSR system response. There are three reasons for the failure of FRSR. First, the sampling ratios of the signals (i.e., 1000/42 ≈ 23.8, 1000/95 ≈ 10.53, 1000/243 ≈ 4.12) are less than 50, which do not meet the large sampling ratio requirement of FRSR. Second, the rescaled characteristic frequencies (i.e., 42/200 = 0.21 Hz, 95/200 = 0.475 Hz and 243/200 = 1.215 Hz) are not small parameters which should be far less than 1. Finally, even though a large enough scale factor (e.g., *R* = 2000) can ensure that the rescaled target signals are small-parameter signals, the output of the FRSR system is divergent. Therefore, when several target signals exist with low sampling ratios, the detection of these signals cannot be realized directly by FRSR. In the next part, the signal $s_{1}\left(t\right)$ will be processed by FERSR.

In order to ensure the comparability of these two methods, the bi-stable system parameters *a* = 0.5, *b* = 4 and the scale factor *R* = 200 are still used for processing the original signal $s_{1}\left(t\right)$ by FERSR. If the reference frequency $f_{l}=8\;\mathrm{Hz}$, then the rescaled reference frequency $f_{l}/R\;=\;8/200\;=\;0.04\;\mathrm{Hz}$ which meets the small-parameter conditions. The sampling ratio of reference low-frequency signal (i.e., 1000/8 = 125) is greater than 50. And the bandwidth of the filters *B* is 32 Hz. According to the analysis in Section 4, a band-pass filter bank *BPFB* and a band-stop filter bank *BSFB* are designed on the basis of the frequencies $f_{1}$, $f_{2}$ and $f_{3}$ correspondingly. While a low-pass filter *LPF* and a high-pass filter *HPF* are designed on the basis of the reference frequency $f_{l}$ respectively.

**Figure 6:**
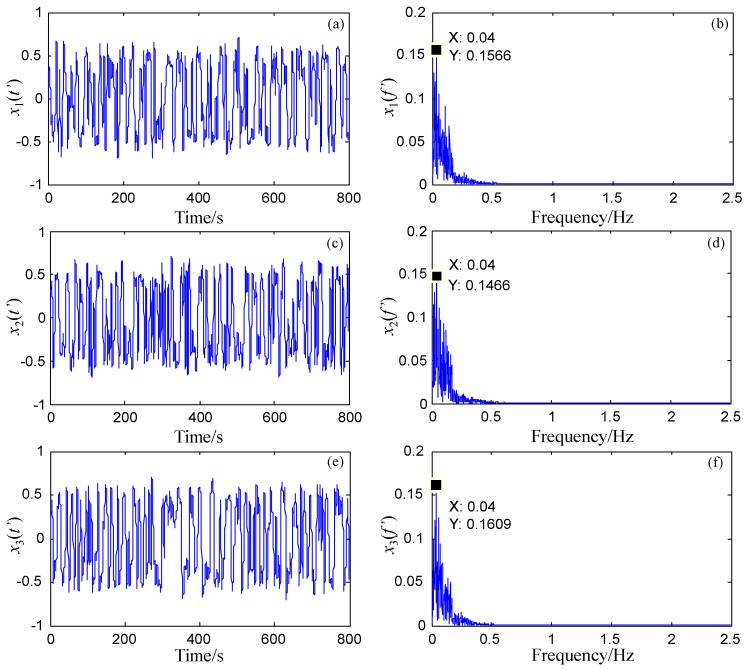
The detection of multi-frequency non-harmonic signal based on FERSR. (**a**,**c**,**e**) are the time-domain waveforms of the response of the FERSR system to the target signals with 42 Hz, 95 Hz, and 243 Hz respectively; (**b**,**d**,**f**) are the corresponding frequency spectra of the response of the FERSR system to the target signals.

Through SSB modulation, the local information around each characteristic frequency extracted by *BPFB* is exchanged with that around the reference frequency $f_{l}$ extracted by *LPF*. The three carrier frequencies are 34 Hz, 87 Hz, and 235 Hz. The responses of the FERSR system to the three exchanged signals are shown in Figure 6. It can be observed that the target signals have obvious spectral peaks at the rescaled reference frequency (0.04 Hz).

**Figure 7:**
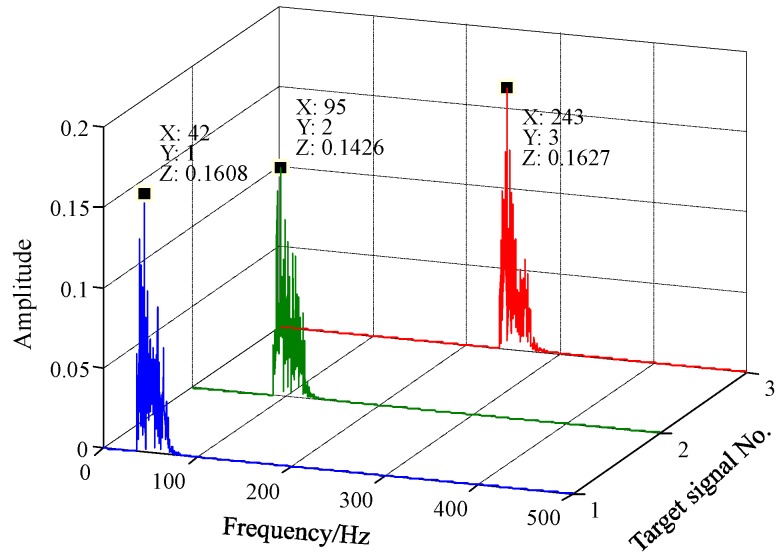
Three-dimensional spectrogram of the multi-frequency non-harmonic signal processed by FERSR and frequency recovery.

In order to determine the presence of the target signals clearly and accurately, the frequency spectra of the responses of the FERSR system to the signals are processed through frequency recovery. As shown in Figure 7, the processed frequency spectra are exhibited in a three-dimensional frequency spectrogram. The multi-frequency non-harmonic signal can be read out from Figure 7. To demonstrate the application of the proposed method, this section presents two case studies concerning the diagnosis of a rolling bearing outer ring fault and a rotor shaft bending fault. The method is used to detect the weak multi-frequency fault signal composed of the first several harmonics of fault characteristic frequency.

### 5.2. Case 1: Application to Fault Diagnosis of Rolling Bearing Outer Ring

**Figure 8:**
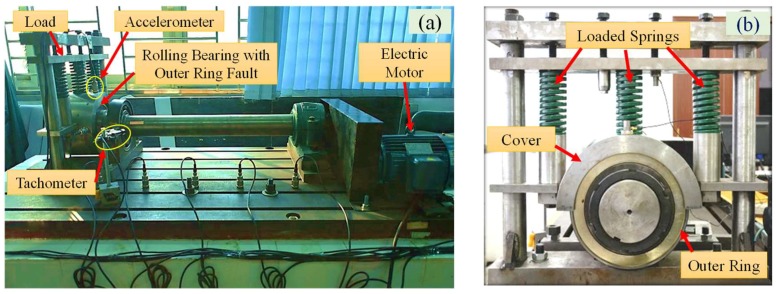
(**a**) The rolling bearing test rig of high-speed train; (**b**) the bearing loading device.

**Figure 9:**
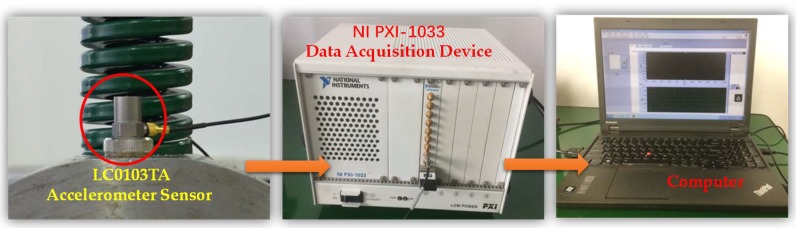
Data acquisition system.

Figure 8a shows the test rig for the rolling bearings of high-speed train. The full load weight of each carriage is 17 t, and every carriage has eight sets of rolling bearings, whose load weight is 2125 kg. To simulate the full-load working conditions of the bearing, the load is applied on the outer ring of the rolling bearing by a semi-circular cover plate which is pressed by a bolt frame structure and three loaded springs, as shown in Figure 8b. The stiffness coefficient of the springs is 300 N/mm. Under full load, the compressed length of the springs is 23.6 mm. The rolling bearing fault detection of the high-speed train is carried out on the test rig and the data acquisition system is shown in Figure 9. Several sensors (LC0103TA accelerometer, Lance Technologies Inc., Buffalo, NY, USA) are fixed on the cover of the rolling bearing and the platform of the test rig. The accelerometer mounted on the cover is used to collect the vibration signals of the rolling bearing. NI PXI-1033 is used to collect data from the accelerometer at a sampling frequency of $f_{s}=1000$ Hz and sampling duration of 10 s.

The fault of the outer ring of the rolling bearing is a common fault, which may have resulted from poor lubrication, foreign matter invasion, and other factors. This kind of fault often occurs through abrasion, pitting and other fatigue failure after long-term use. When defects are formed on the surface of the outer ring (e.g., a pitting fault), the characteristic frequency of the fault can be calculated by the following formula

(25) $$f_{\mathrm{bo}}=\frac{Z}{2}\left(1-\frac{d}{D}\cos\alpha \right)f_{z}$$

where *Z* is the number of rolling elements, *d* is the diameter of the rolling element, *D* is the pitch diameter, α is the contact angle and $f_{z}$ is the rotating frequency of the rotor. According to the parameters of the tested rolling bearing listed in Table 1, the characteristic frequency of the outer ring fault is calculated to be $f_{b0}=7.3\times f_{z}$.

**Figure 10:**
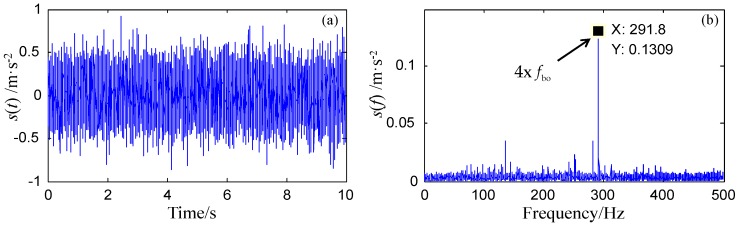
Signals recorded from the rolling bearing of the high-speed train.

In the experiment, the rotating speed is 600 rpm, which means the rotating frequency $f_{z}$ = 10 Hz. Therefore, for an outer ring pitting fault, the frequency $f_{\mathrm{bo}}$ should be close to 73 Hz. Figure 10 shows the original waveform and spectrum of the bearing fault signal. As shown in Figure 10b, except for the fourth harmonic of $f_{\mathrm{bo}}$, its first three harmonics are not able to be recognized because of the strong background noise.

**Figure 11:**
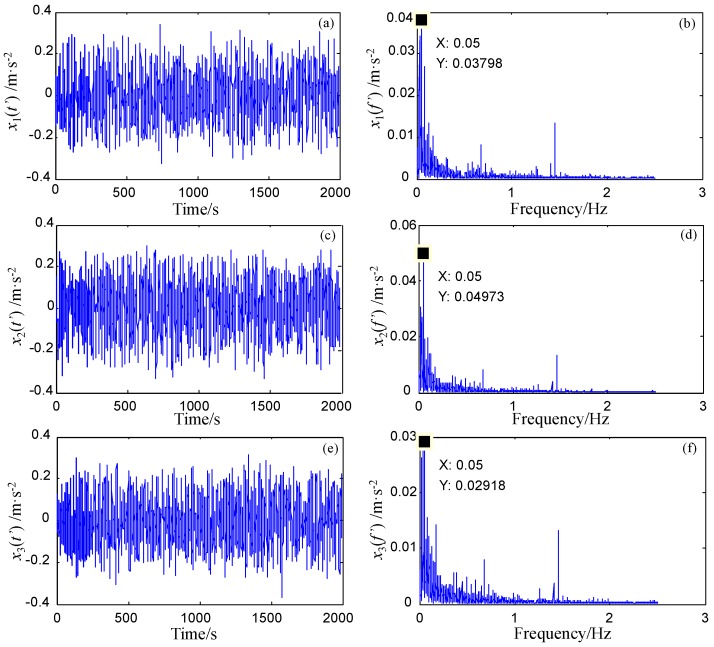
Fault detection of bearing outer ring based on FERSR. (**a**,**b**) are time-domain waveform and frequency spectrum of the detection results of the first harmonic. (**c**,**d**) are that of the second harmonic. (**e**,**f**) are that of the third harmonic.

**Figure 12:**
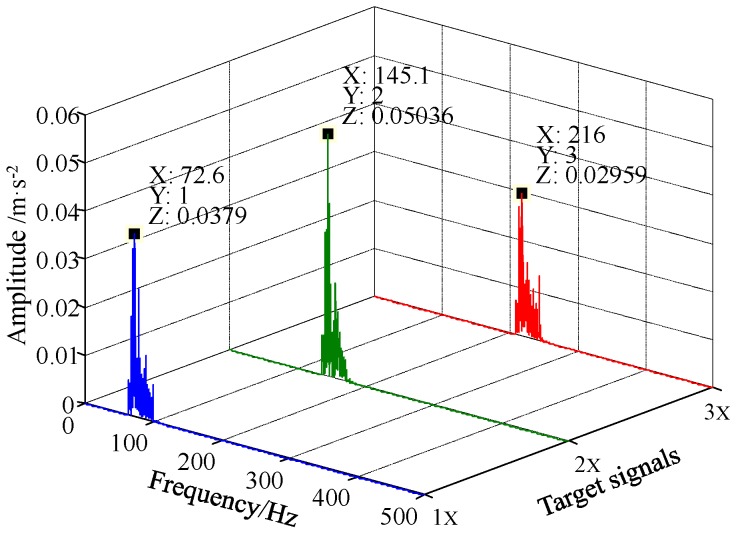
Three-dimensional spectrogram of the bearing outer ring fault signal based on FERSR and frequency recovery.

In this part, the first three harmonics of $f_{\mathrm{bo}}$ are detected by the FERSR method. The sampling ratios of the first three harmonics (i.e., 1000/73 ≈ 13.7, 1000/146 ≈ 6.85 and 1000/219 ≈ 4.57) are less than 50. Setting the reference frequency $f_{l}=10$ Hz and the scale factor *R* = 200, then the rescaled reference frequency is 0.05 Hz which meets the small-parameter conditions. Then the sampling ratio of the reference low-frequency signal (i.e., 1000/10 = 100) is greater than 50. The pass-band width or stop-band width *B* of the filters is set to 20 Hz. The FRSR system parameters are set to *a* = 0.01 and *b* = 4. The detection results of the first three harmonics of $f_{\mathrm{bo}}$ are shown in Figure 11. After frequency recovery, the frequencies of these three target signals are shifted back to their original frequencies, as shown in Figure 12. Obviously, the signals of the bearing pitting fault can be completely identified.

**Figure 13:**
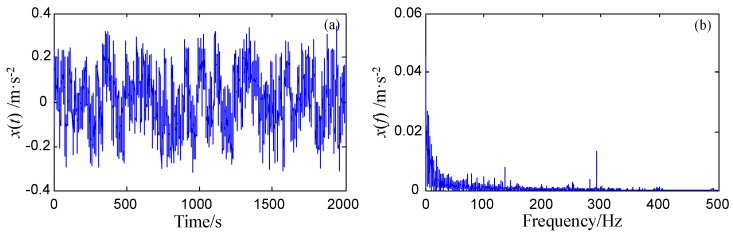
The detection results based on FRSR after frequency scale recovery.

In order to compare the performance of FERSR with that of FRSR, the detection results based on FRSR are also given here. The scale factor and system parameters of FRSR are the same as those of the FERSR method. Figure 13 shows the results of the FRSR system after frequency scale recovery. The first three harmonics of the characteristic frequency cannot be seen clearly in Figure 13, because the rescaled frequencies of the target signals do not meet the small-parameter conditions for the given scale factor *R* = 200. Even though a larger scale factor is selected to ensure the rescaled frequencies meet the conditions, the results of FRSR are divergent and the target signals still cannot be recognized. The reason is that the sampling ratios of the harmonics (13.7, 6.9, and 3.5) are too low to satisfy the sampling ratio requirement of the FRSR method. Hence, this shows that FERSR has an advantage over FRSR in multi-frequency signal detection.

**Figure 14:**
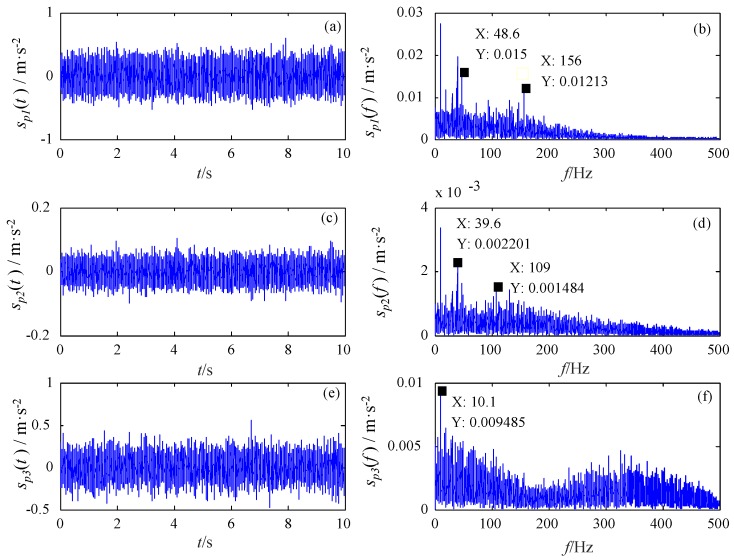
The Singular Value Decomposition (SVD) component signals (**a**,**c**,**e**) and their envelope spectrums (**b**,**d**,**f**).

To further verify the effectiveness of FERSR, the fault diagnosis method based on Singular Value Decomposition (SVD) and envelope analysis is used to extract the weak fault feature from the collected vibration signals. The specific implementation process of this method is given in the reference [38]. The SVD component signals and their envelope spectrums are shown in Figure 14. The fault feature cannot be recognized from the envelope spectrums of the SVD component signals. By comparing the detection results in Figure 12 and Figure 14, it can be concluded that FERSR has better performance in weak fault signal detection.

### 5.3. Case 2: Application to Diagnosis of Rotor Shaft-Bending Fault

**Figure 15:**
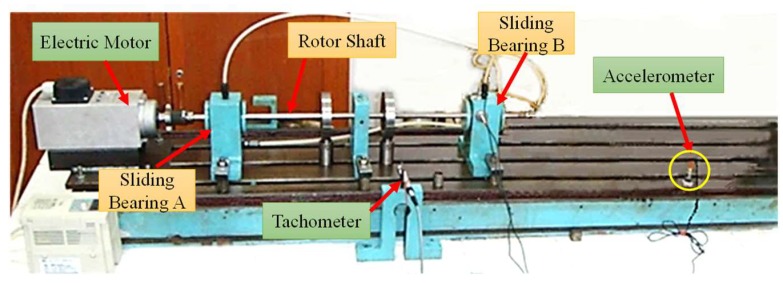
Sliding-bearing test rig for shaft-bending fault experiments.

After long-term running in complex and severe conditions, the shafts of the rotor-bearing systems are usually bent to different degrees, resulting in malfunctions of the systems, which may affect the safe operation of the equipment. The vibration signal of the rotator caused by shaft bending has obvious several harmonics of characteristic frequency. Therefore, attention should be paid to all the features of these components in bending fault detection. Figure 15 shows the sliding-bearing test rig. The diameter of the shaft is *ϕ*12 mm. Due to the deviation of 0.38 mm between the shaft axis and the rotation axis, the shaft-bending and imbalance faults appear in the sliding-bearing rotor system. To simulate the weak fault conditions, the vibration signals of the shaft-bending fault are obtained from an accelerometer (LC0103TA) mounted on an experimental table at a distance of 0.5 m from the bearing base. Through the damping of the bearing and experiment structure, the fault signal components are attenuated, and then recorded by the sensor. In the experiment, the running speed of the rotor is 1680 rpm (i.e., rotating frequency of the shaft is 28 Hz). The data acquisition device NI PXI-1033 is used again to collect data from the sensor at a sampling frequency 1000 Hz and for the duration of 2 s, so that the fault signal has a relatively low sampling ratio.

**Figure 16:**
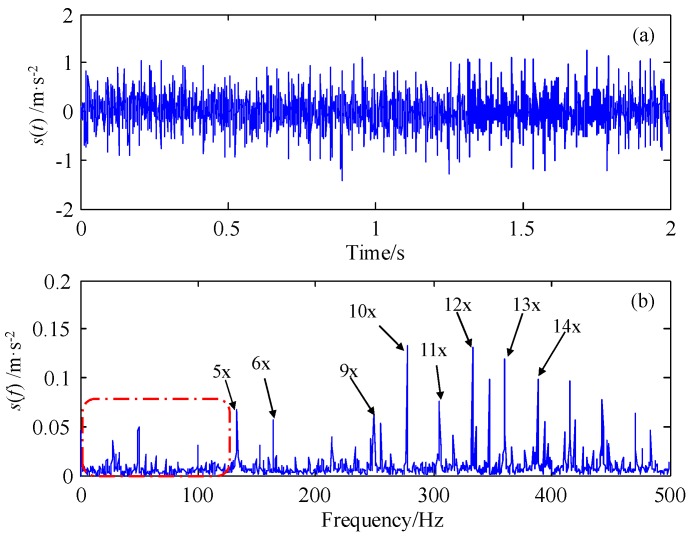
Raw data of the shaft-bending fault (**a**) waveform; (**b**) global spectrum.

Because the sensor is fixed far from the rotor-bearing base, the collected fault signal has been weakened. From the raw data shown in Figure 16, the first four harmonics of the rotating frequency are too weak to be observed, due to the interference from the background noise, higher harmonics, and other irrelevant components. Therefore, it is hard to determine whether the fault exists or not. In order to detect the fault signal and its low-order harmonic components, in the following part, the raw data are processed by the proposed method FERSR.

**Figure 17:**
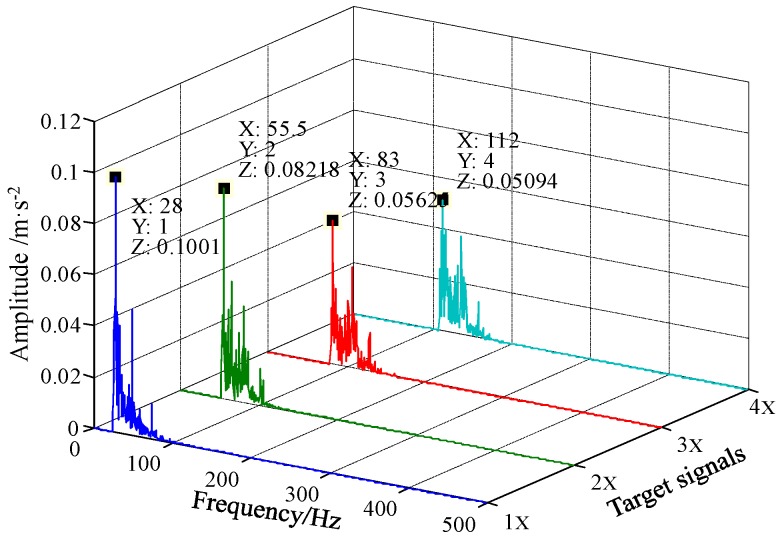
Detection results of the shaft-bending fault based on FERSR and frequency recovery.

First, the first four harmonics of the characteristic frequency are set as target signals. Then the sampling ratios of the first four harmonics (i.e., 1000/28 ≈ 35.7, 1000/56 ≈ 17.85, 1000/84 ≈ 11.9, and 1000/112 ≈ 8.93) are less than 50. The reference frequency is set to 4 Hz and the scale factor *R* is set to 200. Accordingly, the sampling ratio of reference low-frequency signal (i.e., 1000/4 = 250) is greater than 50. And the rescaled reference frequency is 0.02 Hz which meets the small-parameter conditions. The filters used in this case have same stop-band width or pass-band width, i.e., *B* = 20 Hz. The system parameters are *a* = 0.1 and *b* = 10. After processing by FERSR and frequency recovery, the spectra of the four target signals are shown in Figure 17. Therefore, there are obvious spectral peaks which can be identified at the frequencies of the signals (i.e., 28 Hz, 55.5 Hz, 83 Hz, and 112 Hz), so that the presence of the shaft-bending fault of the rotor system can be determined based on FERSR.

### 5.4. Discussion

Although the SR theory has an advantage in weak signal detection, it has still many limitations in the detection of fault signals in some specific systems, such as, rotating machinery systems. On the one hand, the characteristic frequency of the fault signal usually does not meet the small-parameter conditions. On the other hand, the diagnosis of the mechanical fault usually needs to detect the multi-frequency signals composed of the fault signal and its higher harmonic components. However, the existing methods based on SR cannot transform all the characteristic frequencies of the multi-frequency signals into small-parameter frequencies by a single treatment, and many of them are also limited by the sampling ratio of the fault signals.

The FERSR method proposed in this paper overcomes the above-mentioned limitations. Compared to the CSR and FRSR methods, this method has three advantages: (i) FERSR is superior in detecting a weak low-sampling-ratio signal. There is no limitation of sampling ratio for FERSR to process the weak fault signals. That is to say, FERSR has a broader frequency detection range than CSR and FRSR, as shown in Table 2; (ii) The sampling frequency and the number of sampling points needed by FERSR are lower and smaller than that by CSR and FRSR, which makes FERSR more efficient in practical application; (iii) The multi-frequency signal detection method based on FERSR provide a promising tool for fault diagnosis of rotating machines.

The detection result of the simulation shows that FERSR has a better detection performance. Two practical cases demonstrate the feasibility of the proposed approach in practical engineering application. However, the successful application of this method relies on some prior knowledge. It is not so convenient when compared with some adaptive methods. This problem will be solved by introducing some intelligent algorithms into the FERSR method in future work.

## 6. Conclusions

Stochastic Resonance (SR) is a novel phenomenon produced in a nonlinear dynamical system. SR theory can provide a promising tool for weak signal detection and incipient fault diagnosis in engineering applications. However, there are some limitations for the signal detection methods based on SR theory, such as small-parameter condition, high sampling frequency, large data length and so on. To overcome these shortcomings and realize multi-frequency signal detection, the method, Frequency Exchange and Re-scaling Stochastic Resonance (FERSR), was proposed in this paper.

Frequency exchange is carried out in time domain by a filter technique, which can prevent spectrum leakage caused by FFT in the frequency domain. Then, the technique of designing equal-bandwidth filters used in the method can prevent the omission of target signals in the process of frequency exchange. Based on that, a frequency-exchange scheme for multi-frequency signal detection was introduced in Section 4. To verify the reliability and applicability of the proposed method, a simulation and two practical cases were conducted, and the detection results and comparisons analyzed and discussed. The results of analysis show that the FERSR has a better detection performance than other algorithms such as CSR and FRSR. In the future, some intelligent algorithms will be introduced into the FERSR model, to make FERSR more adaptive and useful for weak multi-frequency signal detection and incipient fault diagnosis.

## Figures and Tables

**Table 1:** The main information on the tested rolling bearing.

Pitch Diameter *D* (mm)	Rolling Element Diameter *d* (mm)	Number of Rolling Elements *Z*	Contact Angle α (°)
185	25.25	17	0

**Table 2:** The frequency detection ranges of different methods.

Methods	Frequency Detection Range
CSR	Far less than 1 Hz
FRSR	(0, $f_{s}/50$), $f_{s}$ is sampling frequency
FERSR	(0, $f_{s}/2$)
